# The Personalization-Privacy Paradox in AI-Enabled Health Care: Managing Trust and Governance Under the European Health Data Space

**DOI:** 10.2196/94903

**Published:** 2026-10-27

**Authors:** Maxime Ducret, Julien Cloarec, Nour Alrabie, Mouna Mouncif-Moungache

**Affiliations:** 1 Faculty of Dentistry, Université Claude Bernard Lyon 1, 11 Rue Guillaume Paradin, Lyon, Rhône-Alpes, 69008, France, 33 472722619; 2 Centre de soins dentaires, Pôle d’Odontologie, Hospices Civils de Lyon, Lyon, France; 3 iaelyon School of Management, Université Jean Moulin Lyon 3, Lyon, France; 4 LEREPS, Université Toulouse Jean Jaurès, Toulouse, France; 5 Department of Public Law, Université Jean Monnet, Saint-Étienne, France

**Keywords:** personalization-privacy paradox, European Health Data Space, artificial intelligence, AI, health data governance, privacy calculus, patient trust

## Abstract

AI enables increasingly personalized health care, but the data required intensifies long-standing concerns about privacy, confidentiality, and trust as the European Health Data Space (EHDS) expands the primary and secondary use of health data. This viewpoint examines how the EHDS reshapes the personalization-privacy paradox in AI-enabled health care and under what conditions it can manage, rather than resolve, this paradox. Drawing on privacy calculus theory, we analyze the patient-practitioner relationship, the flow of data from primary to secondary use, and the governance mechanisms introduced by Regulation (EU) 2025/327, including data access bodies, data permits, differentiated opt-out rights, and privacy-preserving technologies. We identify 5 governance conditions: purpose-specific governance, accessible opt-out design, practitioner-mediated transparency, privacy-preserving infrastructure, and independent oversight of secondary use actors. When these conditions are met, the EHDS can sustain trust while enabling personalization. We conclude that the EHDS does not eliminate the paradox but can provide a governance architecture to manage it, depending more on implementation quality and patient engagement than on the ambition of the framework itself.

## Introduction

The personalization-privacy paradox captures a core tension in the digital ecosystem: individuals value the enhanced user experiences that personalization affords, yet remain reluctant to provide the data such personalization requires, expressing concern over how their data are collected, processed, and secured [1]. This paradox has become increasingly salient with the rise of advanced data technologies and AI, which enable refined profiling and predictive analytics [2,3]. AI systems, powered by machine learning and deep learning, can detect patterns in vast datasets and forecast behaviors, needs, and health trajectories, yet this precision amplifies anxieties about surveillance, loss of control, and opaque decision-making. Although personalization is intended to improve engagement and satisfaction, it often provokes unease in consumer and health care contexts [4]. Such concerns are even more critical in health care, where breaches of trust may directly compromise patient safety and clinical outcomes.

In health care, where the General Data Protection Regulation (GDPR) tightly regulates data flows, these tensions are sharper: rules designed to protect patients can also limit the deployment of AI tools with the potential to improve diagnosis, treatment, and prevention [5]. This duality illustrates how personalization can be simultaneously valued and resisted, creating cognitive dissonance for individuals who desire individualized services but remain wary of data exploitation. Academic discourse conceptualizes this ambivalence as the personalization-privacy paradox, emphasizing users’ contradictory attitudes and behaviors [3,6]. To unravel this paradox, scholars have applied privacy calculus theory [2,7-11], which highlights how individuals weigh disclosure trade-offs in exchange for personalization. Well-being has recently emerged as a further determinant, as positive emotional evaluations of digital interactions increase individuals’ willingness to share data [2,3]. Analogous trade-offs, long documented on social media via social exchange theory [8,12-14], now emerge in health care, where patients weigh the benefits of AI-enabled personalization against the risks of misuse, third-party access, or loss of confidentiality. This concern is not uniform across health data: perceived sensitivity varies with diagnosis type, stigma, chronicity, and expected benefit, so patients may readily share routine information while resisting disclosure of information associated with stigmatized or chronic conditions [15-17].

Thus, the personalization-privacy paradox extends beyond individual users to pose a strategic challenge for organizations, which must align consumer psychology with regulatory obligations to design personalization strategies that respect privacy without undermining perceived value [2,18]. The European Health Data Space (EHDS) explicitly addresses both primary health data, collected directly from patients during care, and secondary health data, which refer to the reuse of health information for research, policymaking, or technological innovation [19]. This framework legitimizes and structures these uses, but it also generates tensions between them.

This Viewpoint analyzes the paradox at the individual behavioral level and in relation to institutional and regulatory tensions and applies this framework to the EHDS to derive the conditions under which it can manage, rather than resolve, the paradox. It moves from the patient’s privacy calculus to the practitioner relationship and then to the institutional governance of secondary use. The guiding question of this manuscript is: How can the EHDS manage the personalization-privacy paradox in AI-enabled health care while sustaining patient trust and enabling medical AI innovation?

## The Medical Personalization-Privacy Paradox

The personalization-privacy paradox has often been studied in the domain of human-computer interaction [20]. In health care, however, the paradox acquires greater complexity because personalization is increasingly driven by AI [21]. Unlike in other digital sectors, the stakes in medicine are higher: AI systems are not simply predicting consumer preferences but also influencing diagnoses and treatment pathways, including decisions with major clinical consequences. In this context, the paradox emerges as a tension between protecting patient privacy and harnessing AI to deliver highly personalized care.

In the health care context, “personalization” means adapting diagnosis, treatment, prevention, and monitoring to an individual patient’s biological, genetic, behavioral, and contextual profile, encompassing precision medicine, clinical decision support, risk stratification, and individualized treatment, as distinct from the consumer market sense of targeted content. We further distinguish direct clinical personalization, which tailors care to one patient, from population-level AI analytics that generate aggregate insights. The paradox examined here is most acute in the former: the specific tension between desiring personalized services and resisting the data collection they require, reflecting a broader gap between stated privacy preferences and actual disclosure behavior [22].

Figure 1 illustrates this dynamic through the privacy calculus framework, in which patients’ decisions to share data are mediated by perceived benefits and risks, confidentiality, prior experience, and trust in practitioners and the health care system, as well as by how primary data subsequently flow into secondary use under EHDS governance.

Over the past decade, AI-driven digital health technologies [23] have transformed how medical data are generated, analyzed, and used. Electronic health records (EHRs), wearable sensors, telemedicine platforms, and AI-powered clinical decision support systems now enable continuous data collection and advanced predictive modeling [24]. Machine learning algorithms can, for example, detect early signs of sepsis, personalize oncology treatments, or predict hospital readmission risks with high accuracy. These opportunities for personalization go far beyond traditional models of medical practice. Yet, they also intensify concerns about how patient data are stored, processed, and shared [25].

Historically, health care data exchange has been grounded in the ethical principles of confidentiality and transparent communication, which foster trust between patients and health care professionals. Transparency ensures that patients understand how their information is managed [26]. When responsibly used, AI-driven analytics can help clinicians make earlier diagnoses and tailor treatment, improving clinical outcomes and quality of life [27]. However, the same algorithms often operate as “black boxes,” making it difficult for practitioners to explain how specific predictions or treatment recommendations are generated. This opacity undermines informed consent and challenges traditional notions of trust in the medical relationship. Trust in AI-enabled health care requires both algorithmic explainability (how a model generates its output) and data governance transparency (who accesses data and why). Neither alone is sufficient.

**Figure 1.**
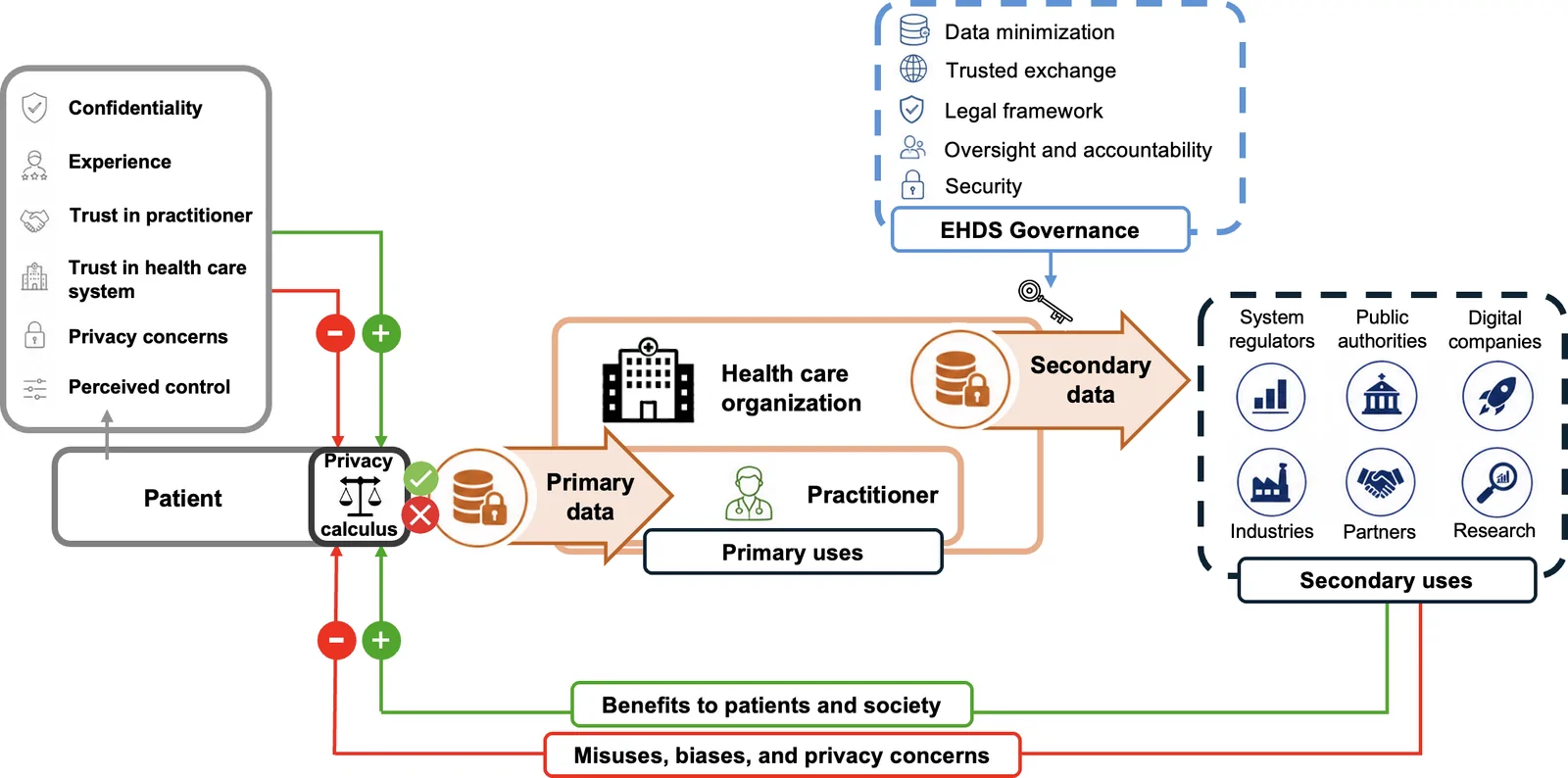
The privacy calculus in the patient-practitioner relationship, the flow of primary data toward secondary use under European Health Data Space (EHDS) governance, and the resulting feedback on patients’ willingness to share data.

A further complication is the increasing involvement of third-party actors, including AI software vendors, private insurers, and digital health app developers, who process patient data outside the traditional patient-health care professional relationship [28]. Trust and acceptance vary markedly across these actors: patients generally trust treating clinicians most, followed by public research institutions and health authorities, with commercial companies and insurers trusted considerably less [29]. Figure 1 illustrates this extension of data flows from practitioners to secondary use stakeholders such as researchers, policymakers, and industry actors, underscoring the risks of commercial exploitation and reidentification.

Pseudonymization has been widely promoted as a safeguard to protect health data [30], and it remains an important safeguard: it reduces direct identifiability and lowers the risk of accidental disclosure. It is not equivalent to full anonymization [31], however, and AI itself heightens the risk of reidentification. Sophisticated algorithms can cross-reference pseudonymized medical records with other datasets (eg, location data, genetic information, and consumer behavior) to reidentify individuals [32]. In health care, where genetic data are inherently unique, this residual risk underscores the need for layered safeguards, combining pseudonymization with access controls, purpose limitation, and privacy-preserving technologies, rather than relying on pseudonymization alone. Within the European Union (EU), the GDPR’s purpose limitation requirements, antidiscrimination law, and the EHDS framework for permitted data use substantially restrict such practices, although reidentification risk warrants continued vigilance.

The medical personalization-privacy paradox is shaped by several interrelated factors, including trust in the health care system [33]. Following Hall et al [34], this analysis distinguishes interpersonal, institutional, and system trust, each requiring different safeguards. Confidentiality has also been a cornerstone of medical ethics since the Hippocratic Oath, serving to foster openness, protect against discrimination, and maintain institutional legitimacy. Yet, AI-enabled personalization is challenging these traditional principles [35]. By aggregating longitudinal health data, including genomics, lifestyle patterns, and real-time biometrics, AI systems magnify both the potential benefits of personalization and the risks of privacy intrusion [36]. Patients face an increasingly difficult calculus: whether to share intimate data with practitioners and institutions that promise better health outcomes but may expose them to unintended risks. Trust in primary use and the governance of secondary use are not independent: when patients discover, or merely suspect, that data shared with their practitioner have circulated to insurers or unfamiliar research institutions, this can retroactively undermine trust in the original clinical relationship and reduce future willingness to share data. The medical personalization-privacy paradox in the age of AI, and its manifestation under the EHDS, can therefore be conceptualized through 5 interdependent dimensions (Table 1).

**Table 1.** Dimensions, opportunities, and challenges of the personalization-privacy paradox within the European Health Data Space (EHDS).

Dimensions	Opportunity	Privacy and ethical challenge	Implication for the paradox
Data requirements and cross-border scale	AI needs vast, multimodal datasets, and EHDS interoperability expands cross-border access, especially for rare diseases.	Larger, cross-border datasets raise the risk of reidentification and expose legal and cultural heterogeneity across member states.	Precision medicine must reconcile data depth and scale with harmonized safeguards.
Transparency and patient awareness	EU^a^ AI Act explainability obligations, combined with EHDS transparency on data access and use, can make AI decision support more interpretable and trustworthy.	Patients see AI as opaque, and practitioners are not always equipped to explain data use.	Trust needs both algorithmic explainability and clear data governance communication.
Patient empowerment and opt-out	Digital platforms and EHDS opt-out give patients differentiated control over data sharing.	Complex consent or opt-out design may exclude less digitally literate patients, skewing AI training data.	Empowerment builds trust but may widen inequity if access is uneven.
Privacy-preserving technologies	Federated learning, homomorphic encryption, and synthetic data enable AI training without centralizing records.	These technologies can be resource-intensive and may reduce performance, while synthetic data and pseudonymization still carry residual risk.	These technologies balance personalization with safeguards but require investment and standardization.
Governance, regulatory, and sectoral tensions	General Data Protection Regulation, EHDS, and the EU AI Act layer protections while enabling responsible innovation across clinical, research, and industry uses.	Oversight may lag behind innovation, and tensions arise when clinical, research, commercial, and insurance actors use data differently.	This dimension requires actor-differentiated, cross-layer governance so AI serves individual and public interests without conflating distinct purposes.

a EU: European Union.

This synthesis illustrates that the personalization-privacy paradox in health care cannot be reduced to a trade-off between convenience and privacy, as in consumer markets. Instead, it reflects a deeper dilemma: whether patients and practitioners can trust AI systems to deliver personalization without compromising the ethical foundation of medicine.

## The EHDS: A New Environment for Patient-Practitioner Data Exchange

The EHDS is a landmark initiative by the EU to create a secure, interoperable framework for health data exchange across member states, with AI-enabled personalization as one particularly salient and privacy-sensitive application among several rather than its defining purpose [37]. It distinguishes between primary use, which involves access for the direct care of the patient concerned, and secondary use, which involves the reuse of health data for research, innovation, policymaking, or regulatory purposes. This distinction structures the governance mechanisms discussed below.

Regulation (EU) 2025/327 establishes mechanisms central to this paradox: Health Data Access Bodies in each member state govern secondary access requests and issue data permits specifying the purpose, scope, and duration of access; patients retain differentiated opt-out rights for primary portability and secondary use; and mandatory interoperability standards ensure consistency in cross-border data exchange. These mechanisms operate within 3 regulatory layers that should not be treated as interchangeable: the GDPR sets the general data protection floor, the EHDS adds a health-specific access and secondary use layer, and the EU AI Act adds risk-based transparency and oversight obligations for high-risk clinical AI.

The EHDS is particularly significant because it situates health data governance at the intersection of health care delivery, digital innovation, and market integration. The EHDS has explicit economic objectives [37]: by facilitating the flow of data across borders, it contributes to the functioning of the internal market and strengthens Europe’s competitiveness in digital health industries.

To achieve these ambitions, the EHDS incorporates robust safeguards aligned with the GDPR and mandates common interoperability standards, seeking to overcome the fragmentation of European health systems while promoting continuity of care and research collaboration.

Nevertheless, the implementation of the EHDS faces significant challenges. Marelli et al [38] argue that the EHDS may be “too big to succeed,” given the risk of fragmented national transposition, disparate data quality, and uneven capacity among access bodies across 27 member states. Ganna et al [39] add that realizing its research potential also depends on data access timelines and investment in federated infrastructure. Governance arrangements will need to be carefully calibrated to prevent fragmented approaches at the national level [40], requiring coordination between EU-wide authorities and those of member states.

## The Challenges of the Personalization-Privacy Paradox in the Context of the EHDS

The EHDS raises new challenges that could exacerbate the personalization-privacy paradox. AI systems in health care require large volumes of sensitive, high-quality data to function effectively, but the very practices that enable personalization may compromise privacy and erode trust [41]. If poorly governed, the EHDS could unintentionally facilitate reidentification, misuse by third parties, or algorithmic biases that harm vulnerable populations [42]. To navigate this tension, it is essential to understand the opportunities and risks of the EHDS in an AI-driven medical context (Table 1).

Three privacy-preserving technologies are particularly relevant here: federated learning, homomorphic encryption, and synthetic data, which are detailed in Table 1. Rare disease research illustrates the stakes: the EHDS’s cross-border pooling of data is essential because no single member state has enough patients to validate AI diagnostic tools, making secondary use for this purpose a clear public interest case, even though it requires sharing data with unfamiliar institutions. Patients are not generally opposed to secondary use: many support it when it serves clear public interest purposes, is governed transparently, and involves trustworthy actors [43,44]. Patient empowerment tools such as consent dashboards and opt-out portals presuppose digital access and health literacy, which are unevenly distributed across EU populations. Consequently, patients with lower levels of digital literacy may be underrepresented or overrepresented in training datasets.

Finally, concerns persist about the potential misuse of data, including discriminatory practices by insurers or the commodification of sensitive health information. AI magnifies these risks: predictive algorithms could be used to deny coverage to high-risk individuals or to market treatments selectively, raising profound ethical dilemmas [45,46]. The long-term success of the EHDS will therefore depend on its ability to sustain public trust by ensuring that the collective benefits of data sharing and AI innovation clearly outweigh the individual risks to privacy. This requires continuous collaboration among stakeholders and vigilant adaptation of regulatory and ethical frameworks to meet the evolving challenges of medical AI.

Taken together, these considerations suggest that the EHDS can manage rather than intensify the paradox when 5 conditions are met: purpose-specific governance separating public interest uses from commercial uses; accessible, user-centered opt-out design; practitioner-mediated transparency; standardized privacy-preserving infrastructure; and independent oversight of secondary use actors. Without these conditions, expanding data access without commensurate safeguards risks deepening rather than resolving the paradox.

## Conclusions

This Viewpoint asked how the EHDS can foster trustworthy AI-enabled personalization while safeguarding patient privacy. The answer is that the EHDS does not resolve the personalization-privacy paradox but, when the 5 conditions identified above are met, provides a governance architecture that can manage it. The future of the paradox will be defined by the EU’s ability to harness AI for transformative health care innovation while preserving the trust that underpins the patient-practitioner relationship.

Success will depend on embedding these conditions into the architecture of AI-enabled health care. Practitioner-mediated transparency and user-centered opt-out design are the most directly actionable conditions in the short term, but the EHDS will offer a replicable model for managing the paradox only if the EU also sustains investment in privacy-preserving infrastructure and independent oversight.

## Abbreviations

EHDS: European Health Data Space
EHR: electronic health record
EU: European Union
GDPR: General Data Protection Regulation
